# Development and testing of diagnostic algorithms to identify patients with acromegaly in Southern Italian claims databases

**DOI:** 10.1038/s41598-022-20295-4

**Published:** 2022-09-23

**Authors:** Salvatore Crisafulli, Andrea Fontana, Luca L’Abbate, Valentina Ientile, Daniele Gianfrilli, Alessia Cozzolino, Maria Cristina De Martino, Marta Ragonese, Janet Sultana, Francesco Barone-Adesi, Gianluca Trifirò

**Affiliations:** 1 Department of Medicine, University of Verona, Verona, Italy; 2 Unit of Biostatistics, Fondazione IRCCS Casa Sollievo della Sofferenza, San Giovanni Rotondo, Italy; 3 Department of Diagnostics and Public Health, University of Verona, P.le L.A. Scuro 10, 37134 Verona, Italy; 4 Department of Biomedical and Dental Sciences and Morphofunctional Imaging, University of Messina, Messina, Italy; 5 Section of Medical Pathophysiology and Endocrinology, Department of Experimental Medicine, Sapienza University of Rome, Rome, Italy; 6 Dipartimento di Medicina Clinica e Chirurgia, Università Federico II di Napoli, Naples, Italy; 7 Department of Human Pathology of Adulthood and Childhood “G. Barresi” DETEV, University of Messina, Messina, Italy; 8 College of Medicine and Health, University of Exeter, Exeter, UK; 9 Department of Translational Medicine, University Piemonte Orientale, Novara, Italy; 10 Research Center in Emergency and Disaster Medicine (CRIMEDIM), University Piemonte Orientale, Novara, Italy

**Keywords:** Endocrinology, Health care, Epidemiology

## Abstract

Acromegaly is a rare disease characterized by an excessive production of growth-hormone and insulin-like growth factor 1, typically resulting from a GH-secreting pituitary adenoma. This study was aimed at comparing and measuring accuracy of newly and previously developed coding algorithms for the identification of acromegaly using Italian claims databases. This study was conducted between January 2015 and December 2018, using data from the claims databases of Caserta Local Health Unit (LHU) and Sicily Region in Southern Italy. To detect acromegaly cases from the general target population, four algorithms were developed using combinations of diagnostic, surgical procedure and co-payment exemption codes, pharmacy claims and specialist’s visits. Algorithm accuracy was assessed by measuring the Youden Index, sensitivity, specificity, positive and negative predictive values. The percentage of positive cases for each algorithm ranged from 7.9 (95% CI 6.4–9.8) to 13.8 (95% CI 11.7–16.2) per 100,000 inhabitants in Caserta LHU and from 7.8 (95% CI 7.1–8.6) to 16.4 (95% CI 15.3–17.5) in Sicily Region. Sensitivity of the different algorithms ranged from 71.1% (95% CI 54.1–84.6%) to 84.2% (95% CI 68.8–94.0%), while specificity was always higher than 99.9%. The algorithm based on the presence of claims suggestive of acromegaly in ≥ 2 different databases (i.e., hospital discharge records, copayment exemptions registry, pharmacy claims and specialist visits registry) achieved the highest Youden Index (84.2) and the highest positive predictive value (34.8; 95% CI 28.6–41.6). We tested four algorithms to identify acromegaly cases using claims databases with high sensitivity and Youden Index. Despite identifying rare diseases using real-world data is challenging, this study showed that robust validity testing may yield the identification of accurate coding algorithms.

## Introduction

Acromegaly is a chronic and progressive endocrine rare disease characterized by an excessive production of growth-hormone (GH) and insulin-like growth factor 1 (IGF-1), which typically results from a GH-secreting pituitary adenoma^1^. It has been estimated that acromegaly globally affects around 6 per 100,000 persons^2^. Epidemiological studies conducted in Italy reported a prevalence of acromegaly ranging from 6.9 to 9.7 cases per 100,000 persons^3–5^. The onset of acromegaly is usually slow and insidious, and the clinical conditions associated with it, such as arthritis, diabetes mellitus, hypertension, sleep apnea and metabolic dysfunction are common in general population^6,7^. As a result, acromegaly is often diagnosed late, thus influencing the long-term disease prognosis^8^ and leading to the development of complications and to increased mortality^9^. Disease prognosis is also conditioned by genetic and epigenetic variants^10^. The recommended diagnostic test for acromegaly consists in the assessment of age/sex normalized IGF-1 levels and GH levels during an oral glucose load in patients with the above-mentioned comorbidities. Nowadays, typical features of acromegaly can also be detected by multimedia tools^11^, useful systems for a modern diagnostic approach. Diagnoses based on laboratory tests should be confirmed thereafter by magnetic resonance imaging (MRI) (or computed tomography scan if MRI is contraindicated or unavailable) which are able to visualize adenoma size and appearance^12^. A multimodal therapeutic approach comprising neurosurgery, medical therapy and radiotherapy is often required to attain biochemical control and reduction of disease-related morbidity and mortality^13^. Surgery is generally recommended as the first-line therapy, while in patients with persistent disease following surgery medical treatment as adjuvant therapy is recommended^14^. There are three pharmacological classes currently available for acromegaly management, to be used as monotherapy or in combination, namely somatostatin analogues (i.e., octreotide, lanreotide and pasireotide), the GH receptor antagonist pegvisomant and dopamine agonists (e.g., cabergoline), which are off-label used^15,16^.

Although acromegaly is a rare disease, the clinical, economic and health-related quality of life burden is considerable due to the wide spectrum of comorbidities and the need for lifelong management^17^. Achieving biochemical control is associated with improvements in cost, quality of life, and mortality, albeit not to the level of the general population^18^.

Claims databases are widely used in pharmacoepidemiology to provide real-world evidence on disease burden, treatment patterns, healthcare resource utilization, benefit-risk profile of drugs and costs^19^. Italy is rich in electronic healthcare data at loco-regional and national level, and these data have been increasingly used in the last decade for research purposes. Large scale distributed database networks have also been set up in Italy, with the potential to substantially increase statistical power when studying rare diseases/outcomes^20^. However, the identification of rare diseases in claims databases remains challenging and requires validated coding algorithms.

Several epidemiologic studies applied different coding algorithms for acromegaly ascertainment in claims databases, but their accuracy has never been assessed, mostly due to the lack of a gold standard cohort with true positive cases.

The aim of this study was to newly develop and revise existing coding algorithms for the identification of acromegaly cases and to compare their accuracy in detecting such patients using claims databases from Caserta Local Health Unit (LHU) and Sicily Region, in Southern Italy.

## Methods

### Data sources

This Italian, retrospective, population-based study was conducted in the period January 2015–December 2018, using data extracted from the fully-anonymized administrative record linkage databases of Caserta Local Health Unit (LHU), with an average of 1,060,904 inhabitants. Moreover, data from the fully-anonymized administrative health databases of Sicily Region in the period January 2011–December 2018, with an average of 5,031,655 inhabitants, were used as a testing dataset to evaluate the performance of the proposed algorithms and to substantiate the estimates of acromegaly cases identified in Caserta LHU. Both databases contain demographic and medical data that is collected through services provided by the Italian National Health Service (NHS). They include information on demographics of residents in each catchment area, outpatient pharmacy claims, hospital discharges, exemptions from co-payment, referrals for outpatient diagnostic tests and specialist’s visits database. The content and context of such NHS claims databases have been described in detail elsewhere^19^. Claims databases from Caserta LHU also comprise the electronic therapeutic plans (ETP) database. In Italy, ETPs are directly filled by the specialists, who provide information on the exact brand name, number of dispensed packages, and indication for use. The dispensed drugs were coded using the Anatomical Therapeutic Clinical (ATC) classification system and the Italian Marketing Authorization Code (AIC), while comorbidities and indications of use were coded through the ninth revision of the International Classification of Diseases—Clinical Modification (ICD-9 CM).

### Algorithm definition

To detect the presence of acromegaly in the target population, four different algorithms were proposed and developed based on a systematic review of published articles of acromegaly epidemiology using claims database-based algorithms^2^. Specifically, each algorithm was developed using a combination of acromegaly-related ICD-9 CM diagnostic codes (253.0), ICD-9 CM surgical procedure codes (07.6x and 92.3x), co-payment exemption codes (001 and 253.0), specialist visits, laboratory exams and pharmacy claims for somatostatin analogues (ATC: H01CB02, H01CB03, H01CB05) or pegvisomant (ATC: H01AX01). “Algorithm 1” was developed by Caputo et al. in 2018^4^, “Algorithm 2” consisted of a revision of the “Algorithm 1”, which included further acromegaly-related codes, while the “Algorithm 3” and the “Algorithm 4” were developed through different combinations of the acromegaly-related codes used in published articles^3,4,21–23^. For each algorithm, as proposed by Caputo et al.^4^, pharmacy claims for drugs approved for acromegaly treatment were not taken into account if (i) patients had received less than three separate drug prescriptions for the treatment of acromegaly (occasional drug users) during the observation period; (ii) the medications were not long-acting release (LAR) formulations; (iii) patients taking octreotide or lanreotide had at least one hospitalization with a diagnosis that, as reported in the summary of product characteristics, is one of the indications for use of these drugs [i.e., malignant neoplasms (ICD-9 CM: 140–209, 230–239), liver disorders (ICD-9 CM: 570–573), gastrointestinal bleeding (ICD-9 CM: 578), esophageal varices (ICD-9 CM: 42), Cushing’s disease (ICD-9 CM: 255; 255.0)]; (iv) patients had a co-payment exemption code for Cushing’s disease (code: 032). Inclusion and exclusion criteria are reported in Table 1.

**Table 1:** Inclusion and exclusion criteria for each proposed algorithm for the identification of acromegaly cases. *Italian coding system.

Algorithm 1	**Inclusion criteria**
	Subjects who had claims suggestive of acromegaly in ≥ 2 of these databases:
	1. Hospital discharge records (ICD-9 CM code: 253.0)
	2. Exemption from co-payment (exemption code: 001)
	3. Prescription or dispensing of any of these drugs: octreotide (ATC: H01CB02), lanreotide (ATC: H01CB03), pegvisomant (ATC: H01AX01), pasireotide (ATC: H01CB05)
	4. Prescriptions for any of the following tests: facial bone nuclear magnetic resonance (88.91.3–88.91.4), cranial computed tomography (87.03–87.03.1)
Algorithm 2	**Inclusion criteria**
	Subjects who had claims suggestive of acromegaly in ≥ 2 of these databases
	1. Hospital discharge records (ICD-9 CM code: 253.0)
	2. Exemption from co-payment (exemption codes: 001, 253.0)
	3. Prescription or dispensing of any of these drugs: octreotide (ATC: H01CB02), lanreotide (ATC: H01CB03), pegvisomant (ATC: H01AX01), pasireotide (ATC: H01CB05)
	4. Prescriptions for any of the following tests: facial bone nuclear magnetic resonance (88.91.3–88.91.4), cranial computed tomography (87.03–87.03.1), 88.97, 90.35.1 (somatotropic hormone measurement), 90.40.6 (IGF-1 levels measurement)
Algorithm 3	**Inclusion criteria**
	1. Hospital discharge records: ≥ 2 diagnostic codes for acromegaly (ICD-9 CM: 253.0) **OR**
	2. Exemption from co-payment: ≥ 1 exemption code for acromegaly (exemption codes: 001, 253.0) **OR**
	3. ≥ 1 prescription or dispensing of any of these drugs: octreotide (ATC: H01CB02), lanreotide (ATC: H01CB03), pegvisomant (ATC: H01AX01), pasireotide (ATC: H01CB05) AND ≥ 1 surgical procedure code for acromegaly (07.6*: Hypophysectomy; 92.3*: Stereotactic radiosurgery) **OR**
	4. ≥ 1 prescription or dispensing of any of these drugs: octreotide (ATC: H01CB02), lanreotide (ATC: H01CB03), pegvisomant (ATC: H01AX01), pasireotide (ATC: H01CB05) AND ≥ 1 prescription for any of the following tests: facial bone nuclear magnetic resonance (88.91.3–88.91.4), cranial computed tomography (87.03–87.03.1), 88.97, 90.35.1 (somatotropic hormone measurement), 90.40.6 (IGF-1 levels measurement)
Algorithm 4	**Inclusion criteria**
	1. Hospital discharge records: ≥ 1 diagnostic code for acromegaly (ICD-9 CM: 253.0) **OR**
	2. Exemption from co-payment: ≥ 1 exemption code for acromegaly (exemption codes: 001, 253.0) **OR**
	3. ≥ 1 prescription or dispensing of any of these drugs: octreotide (ATC: H01CB02), lanreotide (ATC: H01CB03), pegvisomant (ATC: H01AX01), pasireotide (ATC: H01CB05) AND ≥ 1 surgical procedure code for acromegaly (07.6*: Hypophysectomy; 92.3*: Stereotactic radiosurgery) **OR**
	4. ≥ 1 prescription or dispensing of any of these drugs: octreotide (ATC: H01CB02), lanreotide (ATC: H01CB03), pegvisomant (ATC: H01AX01), pasireotide (ATC: H01CB05) AND ≥ 1 prescription for any of the following tests*: facial bone nuclear magnetic resonance (88.91.3–88.91.4), cranial computed tomography (87.03–87.03.1), 88.97, 90.35.1 (somatotropic hormone measurement), 90.40.6 (IGF-1 levels measurement)
**Exclusion criteria (for all algorithms)**	
Pharmacy claims were not taken into consideration if:	
1. Patients had received less than three separate drug prescriptions for the treatment of acromegaly (occasional drug users)	
2. The medications were not long-acting release formulations	
3. Patients taking octreotide or lanreotide had a hospitalization with a diagnosis different from acromegaly, among those for which there is an indication for the use these drugs, as reported in the summary of product characteristics [malignant neoplasms (ICD-9 CM: 140–209, 230–239), liver disorders (ICD-9 CM: 570–573), gastrointestinal bleeding (ICD-9 CM: 578), esophageal varices (ICD-9 CM: 42), Cushing’s disease (ICD-9 CM: 255; 255.0)]	
4. Patients had an exemption code for Cushing’s disease (code: 032)	

### Gold standard cohort definition

In Caserta LHU, gold-standard cases were defined as subjects which had at least one registration in the ETP database with at least one ICD-9 CM code for acromegaly during the study period. Gold-standard non-cases were defined as all remaining subjects (i.e. registered in Caserta LHU with no ICD-9 CM codes for acromegaly in ETP database).

In Sicily Region, gold standard cases were defined as patients with a confirmed diagnosis of acromegaly in the Endocrinology Unit of the University Hospital of Messina, a province that lies in the Northeastern part of Sicily, during the study period. Data concerning non-cases were not available.

### Statistical analysis

For each proposed algorithm, the accuracy was assessed by the sensitivity (SE) and specificity (SP) measures, along with their 95% confidence intervals (CIs) calculated using the exact Clopper–Pearson method for a binomial proportion. Moreover, the Youden Index (i.e. a summary statistic that balances both SE and SP)^24^ was computed and the algorithm that achieved the highest accuracy (i.e. Youden Index) was considered as the preferred one with respect to the others. Furthermore, the positive predictive value (PPV) and the negative predictive value (NPV) along with their 95% CIs, were also estimated to assess the algorithm’s accuracy. To visually assess the overlapping number of acromegaly cases detected by each coding algorithm with respect to different data sources, Venn diagrams were produced.

The four proposed algorithms were also applied to patients diagnosed with acromegaly in the Endocrinology Unit of the University Hospital of Messina through probabilistic record linkage and the proportion of true positives among these cases was compared with the SE achieved for each algorithm. In addition, to provide an estimate of the percentage of acromegaly cases in the Sicilian population, only the algorithm with the highest discriminatory power was performed in the administrative data of the Sicily Region (testing dataset).

Finally, for the algorithm with the highest Youden Index a network plot was produced to show the number of acromegalic patients identified in Caserta LHU and Sicily Region, respectively, to further substantiate the chronological occurrence of any acromegaly-specific code identified in each claims database. In particular, for each claims database, all the possible pathways by which the subjects were identified over time were represented. Each pathway is made of the chronological sequence by which every acromegaly-specific code occurred for each patient. This can be considered a proxy of the patient journey^25^.

All statistical analyses were performed using R Foundation for Statistical Computing (R Development Core Team 2008, Vienna, Austria, version: 4.0.3, packages: *caret* and *PropCIs*).

### Ethics approval

Analyses were conducted in accordance with the ethical standards of the institutional and national research committee and with the 1964 Helsinki Declaration and its later amendments. This study was approved by the Ethics Committee of the *Azienda Ospedaliera Universitaria Integrata* of Verona (Protocol number 55986, 27th September 2021).

### Consent to participate

Informed consent was obtained from all individual patients from the Endocrinology Unit of the University Hospital of Messina included in the study.

## Results

In Caserta LHU, during the period 2015–2018, a total of 84, 92, 116 and 146 individuals were identified as having acromegaly according to “Algorithm 1”, “Algorithm 2”, “Algorithm 3” and “Algorithm 4”, respectively. The estimated percentage of positive cases in Caserta LHU (i.e., the number of detected cases over the total number of subjects) for each proposed algorithm ranged from 7.9 (95% CI 6.4–9.8) to 13.8 (95% CI 11.7–16.2) per 100,000 inhabitants (Table 2).

**Table 2:** Number of subjects identified by each proposed algorithm as having acromegaly (cases) in Caserta Local Health Unit (period January 2015–December 2018) and in Sicily Region (period January 2011–December 2018). CI = confidence interval calculated using the exact Clopper–Pearson method for a binomial proportion.

	Algorithm 1	Algorithm 2	Algorithm 3	Algorithm 4
**Caserta local health unit (study period: 2015–2018)**				
N. identified cases/total population	84/1,060,904	92/1,060,904	116/1,060,904	146/1,060,904
N. identified cases per 100,000 inhabitants (95% CI)	7.9 (6.4–9.8)	8.7 (7.1–10.6)	11.0 (9.1–13.1)	13.8 (11.7–16.2)
**Sicily region (study period: 2011–2018)**				
N. identified cases/total population	394/5,031,655	533/5,031,655	768/5,031,655	824/5,031,655
N. identified cases per 100,000 inhabitants (95% CI)	7.8 (7.1–8.6)	10.6 (9.7–11.5)	15.3 (14.2–16.4)	16.4 (15.3–17.5)

**Figure 1:**
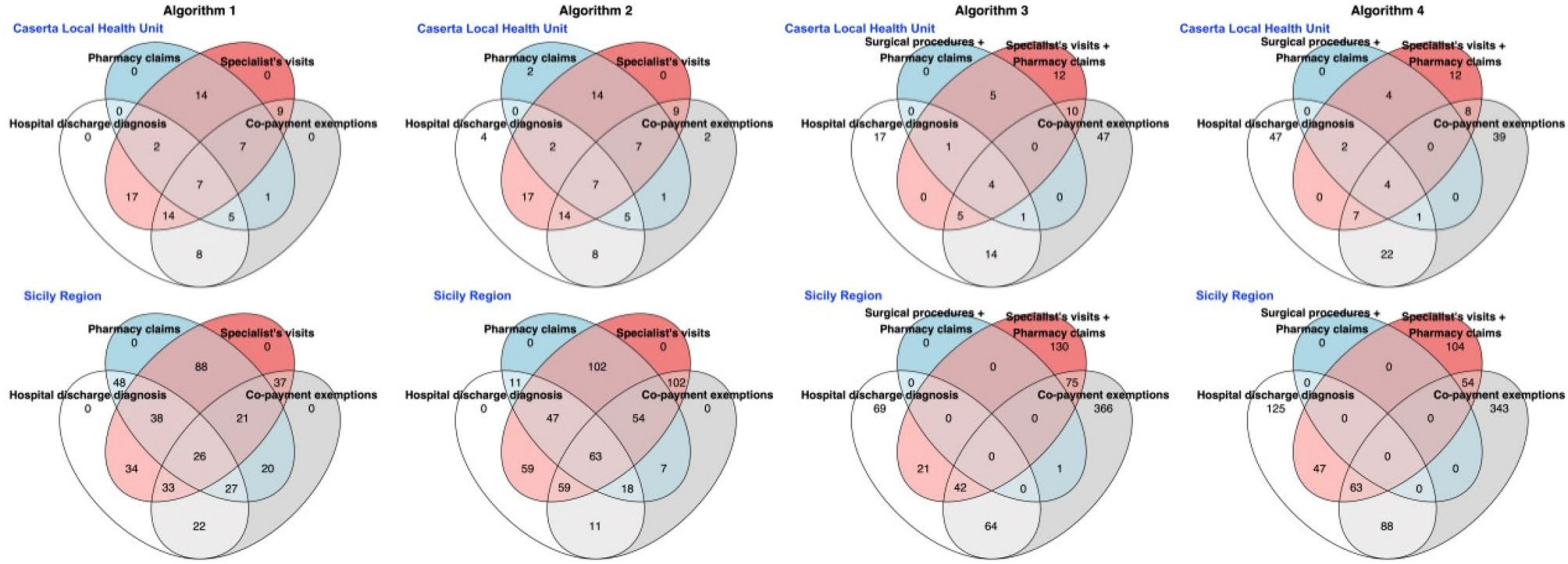
Frequency distribution (Venn diagrams) of the number subjects identified by each proposed algorithm as having acromegaly, with respect to different data sources (i.e. databases of Caserta Local Health Unit and Sicily Region) during the study period.

Seven overlapping cases were detected at all data sources by “Algorithm 1” and “Algorithm 2”, whereas 4 overlapping cases were detected in all data sources by “Algorithm 3” and “Algorithm 4”, respectively (Fig. 1).

Of the 1,060,904 subjects registered in Caserta LHU, 81,388 (7.7%) had least one registration in the ETP database. Among them, 38 individuals with at least one ICD-9 CM code for acromegaly (i.e. gold-standard cases) were identified, yielding an estimated prevalence of 3.6 (95% CI 2.6–4.9) cases per 100,000 inhabitants. The mean age of gold-standard cases was 54.5 ± 13.9 years and 20 (52.6%) of them were females. Concerning the 1,060,866 gold-standard non-cases, their mean age was 26.6 ± 20.7 years and 538,086 (50.7%) of them were females.

For each proposed algorithm, the number of true and false positive/negative counts as well as diagnostic measures are reported in Table 3.

**Table 3:** Number of subjects with at least one diagnosis code for acromegaly in the electronic therapeutic plans databases from Caserta Local Health Unit (cases) and other subjects registered in Caserta Local Health Unit (non-cases) in the period January 2015–December 2018, as well as number of true and false positive/negative counts, diagnostic and predictive accuracy measures with respect to each proposed algorithm. ^PPV and NPV were computed taking into account that the disease prevalence in Caserta Local Health Unit was 3.61 cases per 100,000 persons (i.e. 38 true cases over a total sample of 1,051,943 subjects); °CI calculated using the exact Clopper–Pearson method for a binomial proportion; ^§^CI calculated using the standard logit confidence intervals. Predictive measures are dependent on disease prevalence; *The Youden Index is computed as the sum of SE (%) and SP (%) minus 100% and denote the accuracy of each considered algorithm; ^#^The algorithm with the highest accuracy. *TP* True Positive, *FP* False Positive, *FN* False Negative, *TN* True Negative, *SE* sensitivity, *SP* specificity, *PPV* Positive Predictive Value, *NPV* Negative Predictive Value, *CI* confidence interval.

	Counts								Accuracy measures (95% CI°)		Youden index (%)*	Predictive accuracy measures (95% CI^§^)	
	TP	FP	FN	TN	Cases	Non-cases	Positive	Negative	SE (%)	SP (%)		PPV (%)^	NPV (%)^
					(TP + FN)	(FP + TN)	(TP + FP)	(TN + FN)					
Algorithm 1	27	57	11	1,060,809	38	1,060,866	84	1,060,820	71.05 (54.10–84.58)	99.995 (99.99–100.00)	71.047	32.1 (25.4–39.7)	99.999 (99.999–100)
Algorithm 2	32	60	6	1,060,806	38	1,060,866	92	1,060,812	84.21 (68.75–93.98)	99.994 (99.99–100.00)	84.205^#^	34.8^#^ (28.6–41.6)	99.999 (99.999–100)
Algorithm 3	30	86	8	1,060,780	38	1,060,866	116	1,060,788	78.95 (68.68–90.45)	99.992 (99.99–100.00)	78.939	25.9 (21.1–31.3)	99.999 (99.999–100)
Algorithm 4	32	114	6	1,060,752	38	1,060,866	146	1,060,758	84.21 (68.75–93.98)	99.989 (99.99–100.00)	84.200	21.9 (18.2–26.1)	99.999 (99.999–100)

SE ranged from 71.1% (95% CI 54.1–84.6%) for “Algorithm 1” to 84.2% (95% CI 68.8–94.0%) for “Algorithm 2” and “Algorithm 4”, while SP was higher than 99.9% for each proposed algorithm. The “Algorithm 2” achieved both the highest accuracy (i.e. Youden Index of 84.2%) and the highest accuracy (PPV of 34.8; 95% CI 28.6–41.6) and it was designated to be the preferred one.

The application of the coding algorithms on the claims databases of Sicily Region yielded a total of 394, 533, 768 and 824 acromegaly cases identified by “Algorithm 1”, “Algorithm 2”, “Algorithm 3” and “Algorithm 4”, respectively, with an estimated percentage of detected cases ranging from 7.8 (95% CI 7.1–8.6) to 16.4 (95% CI 15.3–17.5) per 100,000 inhabitants (Table 2). Only twenty-six and sixty-three overlapping cases were detected at all data sources by the “Algorithm 1” and “Algorithm 2”, respectively (Fig. 1). When assessed on the 76 (46 females and 30 males) confirmed acromegaly cases in the Endocrinology Unit of the University Hospital of Messina (Sicily), the percentage of true positives detected by “Algorithm 2” was 82.9% (95% CI 72.5–90.6), which was quite consistent with SE estimate in the Caserta LHU database. The mean age at diagnosis of these patients was 47.7 ± 17.4 years.

**Figure 2:**
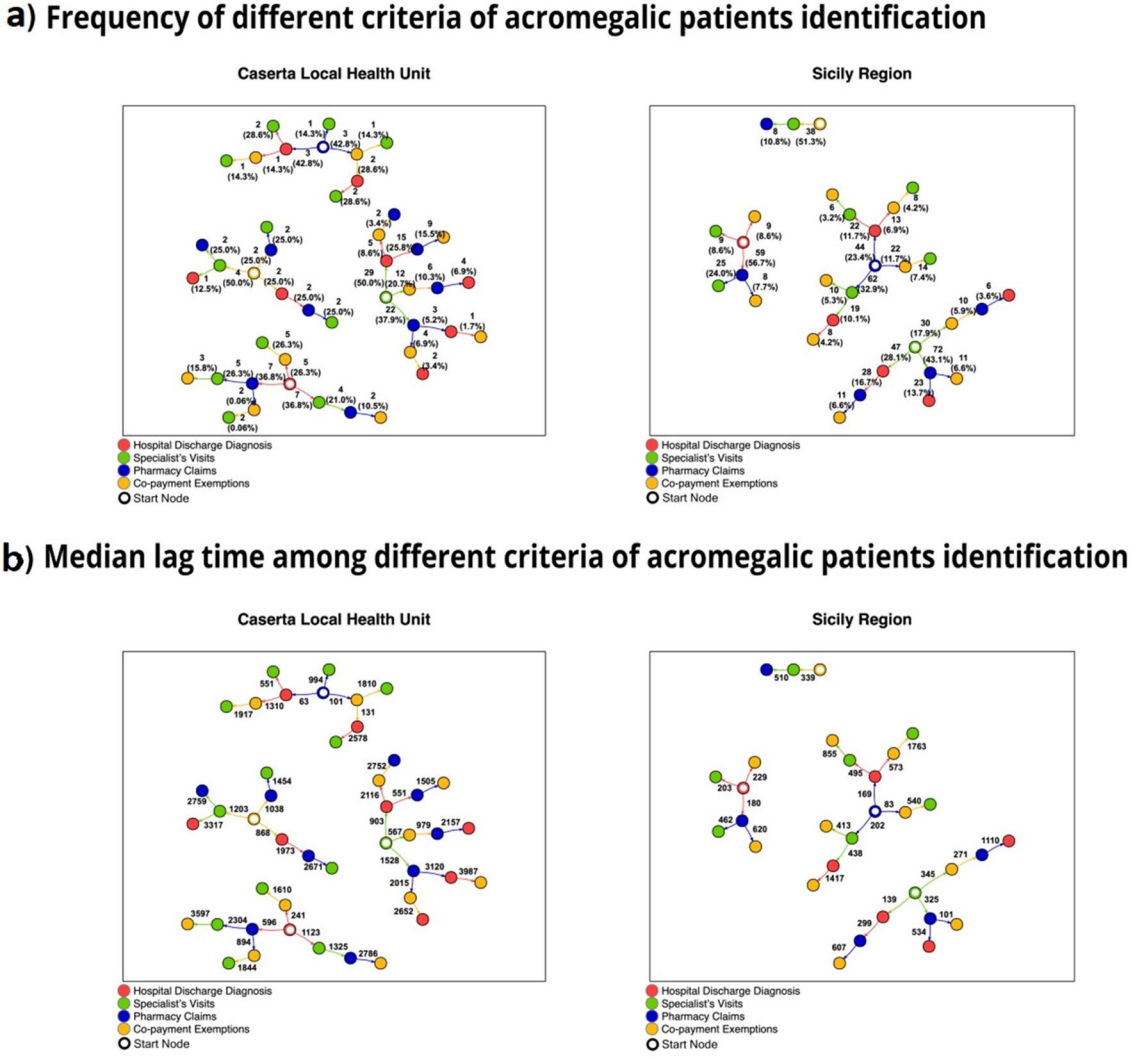
Network plots showing the chronological occurrence of each acromegalic-specific code included in the algorithm 2 in both Caserta Local Health Unit and Sicily Region. Each node represents each claims database (i.e., diagnostic codes, co-payment exemption codes, specialist’s visits and pharmacy claims). Edges define the chronological direction to follow to identify a specific pathway. In panel (a) the frequencies (defined with respect to the number of patients in the start node) of different criteria of acromegalic patients’ identification are reported along the edges. For instance, starting from the specialist visit records in Caserta Local Health Unit, 12 patients (20.7%) having both a first acromegaly diagnosis recorded in that dataset plus a later additional acromegaly-specific code in the co-payment exemptions database were counted. In panel (**b**) the corresponding median lag time (days) among different criteria of acromegalic patients’ identification is reported for each pathway identified. For instance, starting from the specialist visit records in Caserta Local Health Unit, for the 12 patients (20.7%), a median time of 567 necessary days to be identified also by a later additional acromegaly-specific code in the co-payment exemptions database was observed.

Patient journey maps showed that, for the majority of acromegalic patients identified by the “Algorithm 2” in Sicily Region, the first acromegaly-related code occurred in pharmacy claims database (188/533; 35.3%), while in Caserta LHU the first acromegaly-identifying code was mostly found in specialist’s visits database (58/92; 63.1%). The pathway of chronological occurrence of acromegaly-related codes in each claims database is shown in Fig. 2.

## Discussion

To our knowledge, this is the first population-based study that assessed and compared the accuracy of different coding algorithms for the identification of patients with acromegaly using claims databases. Generating epidemiological evidence on rare diseases like acromegaly is crucial to evaluate their impact in terms of burden of disease and to identify unmet clinical needs.

All the four algorithms tested were able, to different extent, to identify true acromegalic patients in claims databases. Based on this, we found that both “Algorithm 2” and “Algorithm 4” provided the most reliable results matching the majority of true acromegalic patients (84.2%). In particular, the preferred “Algorithm 2” achieved the highest discriminatory accuracy, as shown by the Youden Index and PPV, and yielded an estimated prevalence comparable to the global one estimated in the meta-analysis^2^. Furthermore, as compared to the “Algorithm 4”, “Algorithm 2” yielded a better performance also according to principle of parsimony, since it required less acromegaly-identifying codes to be computed, achieving the same SE and higher SP. This algorithm was developed from the one proposed by Caputo et al.^4^ (“Algorithm 1”) by adding further codes for specific laboratory exams, such as the codes for somatotropic hormone measurement and IGF-1 levels measurement, and searching for acromegaly-specific co-payment exemptions also using ICD-9 codes. Indeed, in Italy, it is not uncommon that disease-specific co-payment exemptions are registered using the ICD-9 code rather than the specific exemption code. As compared to “Algorithm 1”, the addition of these codes led to considerable improvements in their accuracy.

The estimated percentages of positive acromegaly cases identified using both Caserta LHU and Sicily Region claims databases were slightly higher than the global prevalence estimated in our meta-analysis of observational studies [5.9 (95% CI 4.4–7.9) cases per 100,000 persons]^2^, but they were comparable to those reported in the Italian epidemiological studies, conducted by Gatto et al.^3^ and Caputo et al.^4^. This corroborates the fact that prevalence estimates based on the proposed algorithms are consistent when applied to different databases of different Italian geographical areas.

One of the main obstacles in using real-world data sources, especially for rare diseases, is related to disease coding and to the availability of specific diagnostic codes. In many cases, rare diseases do not have specific codes (e.g. muscular dystrophies) nor co-payment exemption codes^20^. However, as compared to other rare diseases, acromegaly diagnostic (ICD-9 CM: 253.0) and co-payment exemption (001) codes are rather specific, even if they also refer to gigantism, a clinical condition due to GH overproduction during childhood and adolescence, specifically. Therefore, such codes are reliable enough to identify acromegaly in claims databases. This also holds true for the drugs used for the management of acromegalic patients. The GH receptor antagonist pegvisomant is exclusively approved for the treatment of acromegaly in patients who have had an inadequate response to surgery and/or radiation therapy and/or other medical therapies; although somatostatin analogues are also approved for indications other than acromegaly, they are reliable proxies of acromegaly, especially when used in combination with other acromegaly-related codes.

Patient journey maps showed that most of the cases were primarily identified through pharmacy claims and specialists’ visit. Accordingly, the frequency of those healthcare services is in general much higher than hospitalizations, surgical procedures and exemption codes and, as compared to other services, are more likely to be more accurately identified in claims databases.

One of the main strengths of this study is that it is a large-scale population-based study, for a total of more than 6,000,000 patients from both Caserta LHU and Sicily Region. This is especially relevant for research in the field of rare diseases, where the number of affected patients is very small. However, some limitations warrant caution. Considering that the database used was claims-based, this study presents some limitations associated to this kind of data sources, such as potential misclassification due to potentially inconsistent and inaccurate coding practice. Moreover, since claims data are primarily collected for administrative purposes (e.g., reimbursement), the proposed coding algorithms may yield different performances in populations with different administrative practices and coding patterns. Finally, given that complete remission from the disease is achieved in around 50% of surgically-treated patients^26–30^ and that medical therapy is mainly indicated in those patients who failed to achieve remission after surgery, the extraction of the gold standard cohort from the ETP database may not be fully representative of acromegaly cases. Nevertheless, this has unlikely affected the comparison of the accuracy of different coding algorithms for acromegaly identification.

## Conclusions

In this study, we have developed and tested four algorithms for the identification of acromegaly using claims databases from Caserta LHU and Sicily Region, achieving highly consistent diagnostic accuracy measures. Despite identifying a rare disease such as acromegaly using real-world data is challenging, this study showed that robust validity testing may yield the identification of accurate coding algorithms.

## Data Availability

The individual patient-level dataset generated and/or analyzed during the current study is not publicly available due to the agreement with data provider, but aggregated data can be shared from the corresponding author on reasonable request.
